# Citrate of Iron and Sulphate of Quinine in Chlorosis and Other Anœmic States

**Published:** 1846-05-01

**Authors:** 


*Citrate of Iron and Sulphate of Quinine in Chlorosis and other Anæmic States.* By Dr. Meigs. (Lond. Med. Gaz., Sept., 1845.)—I have made most advantageous use of a citrate of iron, conjoined with sulphate of quinia, as in the following formula, which I subjoin as a very convenient and successful one in the disorders dependent upon anæmia. Take of citrate of iron 2 drachms; sulphate of quinia ½ drachm; water 1 fluid ounce. Mix, and direct from 20 to 30 drops for the dose, in syrup and water. Agreeably to M. Raciborski's method, I advise the patient to take the draught after each meal, within half an hour of the breakfast, dinner or supper, so that it may be carried with the chyme along the course of the bowels. The addition of the quinia to the ounce of water renders the citrate and the sulphate perfectly soluble, as does also the addition of a few drops of ammonia to a solution of the citrate alone; without some addition, a part of the citrate of iron is not dissolved.

